# Time as a Therapeutic Ally: The Promise of Long-Term Solid Organ and Tissue Perfusion

**DOI:** 10.3389/ti.2026.16100

**Published:** 2026-04-17

**Authors:** Florian Huwyler, Matthias Pfister, Diwakar Phuyal, Yomna E. Dean, Margareta Mittendorfer, Lars Saemann, Hannah Rasel, Simon Stoerzer, Jonas Binz, Bahareh Tabatabaei, Gabor Szabo, Sandra Lindstedt, Bahar Bassiri Gharb, Mark W. Tibbitt, Pierre-Alain Clavien

**Affiliations:** 1 Hub for Translational Research and Liver/GI Health, Zurich, Switzerland; 2 Macromolecular Engineering Lab, ETH Zurich, Zurich, Switzerland; 3 Department of Surgery and Transplantation, University of Zurich, Zurich, Switzerland; 4 Department of Plastic and Reconstructive Surgery, Cleveland Clinic Foundation, Cleveland, OH, United States; 5 Department of Clinical Sciences, Lund University, Lund, Sweden; 6 Department of Cardiothoracic Surgery and Transplantation, Skåne University Hospital, Lund, Sweden; 7 Department of Cardiac Surgery, University Hospital Halle, Halle, Germany

**Keywords:** heart, kidney, liver, lung, transplant, vascularized composite allografts

## Abstract

Rapid advances in tissue preservation and the growing adoption of machine perfusion have fundamentally reshaped solid-organ and tissue transplantation in recent years. Multiple short-term perfusion devices have received regulatory approval and are increasingly used in clinical practice to preserve grafts for several hours, improving allograft assessment. The boundaries of dynamic tissue preservation have been pushed even further in research settings, where grafts have been reliably perfused for multiple days. The extended time of long-term machine perfusion opens a new therapeutic window for interventions, allowing for reconditioning and even tissue repair of injured and diseased grafts. The increasing global organ shortage makes these approaches particularly attractive to recover additional allografts for safe transplantation. In this review, we highlight current clinical practice for *ex situ* perfused allografts, multi-day perfusions in research settings, and potential therapeutic benefits of long-term perfusion with a focus on hearts, livers, lungs and vascularized composite allografts.

## Introduction

### Global Organ Shortage

Solid-organ transplantation has been successfully established since the first transplantation of a human kidney in 1954 [1]. Since then, heart, lung, liver and kidney transplantation has become the only sustainable therapy for patients with end-stage organ diseases [2]. Further, transplantation of vascularized composite allografts (VCAs), including upper and lower extremities, face, abdominal wall, uterus and penile grafts, has become an available option to recover the quality of life for patients [3, 4]. However, due to increasing demands for allografts, extension of transplant indications, and increasing prevalence of organ diseases in the general population, we face a global shortage of transplantable grafts. At the beginning of 2025, 13,570 patients were awaiting an organ transplantation in the Eurotransplant-area [5], and more than 100,000 in the US [6]. Therefore, strategies are urgently needed to increase utilization of available grafts safely. To address this gap, transplant surgeons have already started extending acceptance criteria by using marginal grafts, including those from aged donors and grafts that were donated after circulatory arrest (DCD). Because these grafts are associated with higher risk of non-anastomotic complications when they are transplanted without being evaluated or resuscitated on a perfusion device, researchers have developed dynamic preservation platforms that allow for graft assessment and reconditioning prior to transplantation [7–9].

### Dawn of Dynamic Preservation

Current perfusion concepts all share the core function of supplying oxygen to the donor graft while outside of the body, but differ in terms of temperature, perfusate composition, and time of perfusion [10]. Three core concepts of dynamic preservation have gained increasing acceptance across most allografts: *ex situ* perfusion in normothermic (35 °C–38 °C) versus hypothermic (4 °C–12 °C) conditions, and *in situ* normothermic (35 °C–38 °C) regional perfusion (NRP) [11, 12].

Normothermic machine perfusion (NMP) is generally applied either immediately after procurement and during transport, which limits the period allografts are exposed to cold ischemia, or after an initial phase of cold storage, referred to as « back-to-base», at the transplant center (end-ischemic NMP) [10]. NMP typically utilizes an oxygenated blood-based perfusate, either through blood products or donor whole blood, which is pumped through afferent vessels of the allografts. The high demands of metabolically functional allografts necessitate sophisticated perfusion machines that closely mimic the physiological environment *in situ* [13]. Acellular perfusates with synthetic oxygen carriers have also been used for NMP to avoid demand for blood products and overcome issues related to hemolysis, but these approaches remain the subject of preclinical research [14, 15]. NMP has already been leveraged to perform graft assessment, evaluating parameters, such as bile production in livers [16], weight change in VCAs [17], monitoring oxygenation in lungs [18] or coronary flow and resistance in hearts [19]. While such objective assessment has increased utilization of marginal grafts [20], consensus on biomarkers and their reliability has yet to be established.

In an alternative approach, hypothermic oxygenated perfusion (HOPE) mainly relies on chilled synthetic preservation solutions, typically containing osmotic agents, electrolytes, buffering substances, metabolic substrates, and antioxidants or free radical scavengers [21, 22], that are pumped through afferent vessels at 4 °C–8 °C [23]. In livers, perfusate may further be administered through the portal vein only (HOPE) or both, the portal vein and the hepatic artery (dual or D-HOPE), with similar long-term outcomes [24]. Hypothermic conditions markedly decrease metabolic demands, allowing allografts to rely only on dissolved oxygen in the perfusate without the need for oxygen carriers in the perfusate. Therefore, hypothermic perfusion machines are inherently simpler and less costly than their normothermic counterparts [25]. Core functions include perfusate cooling and oxygenation as well as maintaining a steady perfusate flow. The main advantage of HOPE—typically applied in an end-ischemic setting—lies in restoring aerobic metabolism at lower temperatures, thus reducing toxic metabolite accumulation (i.e., succinate, NADH) and subsequent reactive oxygen species production, with the aim of dampening ischemia–reperfusion injury (IRI) [26–28]. While HOPE, like NMP, allows for the measurement of biomarkers to predict allograft viability [29–31], assessment of allograft function during preservation remains limited in clinical practice.

NRP is initiated immediately after circulatory death with the intention of avoiding prolonged ischemia in the organ procurement process prior to reperfusion—a concept particularly appealing in donation after cardiac death (DCD) and standard practice in several countries, including Italy, Spain, France, and parts of the United Kingdom [32]. Both the abdominal compartment alone and thoraco-abdominal organs combined may be included in NRP circuits [33, 34]. NRP includes clamping either the thoracic aorta or ligating the cerebral vessels to prevent cerebral blood flow [33]. Common extracorporeal membrane oxygenation (ECMO) devices are employed to generate artificial blood flow [35]. This approach offers the earliest opportunity to assess allograft viability, though reflecting a multi-organ environment [32]. While promising from an allograft utilization perspective, this technique still faces ethical and legal barriers related to the *dead donor rule* in many countries [36–38]. Hence, NRP will not be discussed in detail in this article.

## Clinical Use of Short-Term Perfusion

Dynamic organ preservation has transformed transplantation across multiple organs, with accumulating evidence demonstrating superiority over static cold storage. In liver transplantation, *ex situ* machine perfusion technologies—including four RCTs for NMP [39–42] and six for HOPE [43–48] —have shown improved graft survival, reduced post-operative and liver-related complications, and decreased ischemic cholangiopathy in donation after circulatory death (DCD) transplantation (Table 1). Machine perfusion further improved utilization rates enabling safe use of previously declined donor livers through enhanced viability assessment [9, 20, 29, 30, 67–70]. Current research explores complementary combinations including use of controlled oxygenated rewarming (COR), combinations like HOPE-COR-NMP [71–75], NRP-HOPE [76–78], and NRP-NMP [79, 80] sequences, marking the end of static cold storage as standard practice [81]. However, NRP, which is praised for improving organ utilization and its protective effect on the biliary tree [80, 82–84], has not been studied prospectively to date.

**TABLE 1:** Randomized controlled trials for the use of machine perfusion in transplantation of livers, hearts, lungs, and kidneys.

Graft type	Study (year)	N (total transplants)	Graft type (perfusion group)	Perfusion type	Median perfusion duration (h)	Control	Primary endpoint
Liver	Nasralla [39]	220^a^	37.1% DCD	NMP	9.1	SCS	Peak AST in 7 days
	Markmann [40]	298^a^	19% DCD	NMP	4.6	SCS	EAD
	Ghinolfi [42]	20	DBD	NMP	4.2	SCS	6-month graft/patient survival
	Chapman [41]	266^a^	14.3% DCD	NMP	5.9^b^	SCS	EAD
	Schlegel [44]	170^a^	DBD	HOPE	1.6	SCS	Patients with Clavien ≥ III
	Panayotova [45]	179	8% DCD	HOPE	2.8	SCS	EAD
	Van Rijn [43]	160	DCD	HOPE	2.25	SCS	6-month non-anastomotic biliary strictures
	Czigany [46]	46	DBD	HOPE	2.4	SCS	Peak ALT after 7 days
	Grat [48]	104	DBD	HOPE	2	SCS	Model for early allograft function
	Ravaioli [47]	110	DBD	HOPE	2.4	SCS	EAD
Heart	Ardehali [49]	130	DBD	NMP	3.5	SCS	30-day patient and graft survival
	Schröder [50]	180	DCD	NMP	Not reported	SCS, DBD grafts	6-month survival
Lung	Slama [51]	80	DBD	NMP	4.4	SCS	Pao_2_/Fio_2_ ratio, primary graft dysfunction after 24h
	Warnecke [52]	320	DBD	NMP	3.7	SCS	30-day survival, absence of primary graft dysfunction grade 3 after 72h
Kidney	Moers [53]	672	12.5% DCD	HMP	15^c^	SCS	Delayed graft function
	Wang [54]	48	DCD	HMP	6	SCS	Delayed graft function
	Malinoski [55]	1349	DBD	HMP	19.3^c^	SCS	Delayed graft function
	Husen [56]	262	DBD	HMP	4.7	SCS	1-year graft survival
	Alijani [57]	58	DBD	HMP	32.5^c^	SCS	Delayed graft function
	Halloran [58]	181	DBD	HMP	30.5	SCS	1-year graft survival
	Merion [59]	100	DBD	HMP	1	SCS	Delayed graft function and post-transplant serum creatinine levels
	Summers [60]	102	DCD	HMP	13.9^c^	SCS	Delayed graft function
	Tedesco-Silva [61]	160	DBD	HMP	25.05^c^	SCS	Delayed graft function
	Van der Vliet [62]	76	DCD	HMP	Not reported	SCS	Delayed graft function and primary nonfunction
	Watson [63]	80	DCD	HMP	10.1	SCS	Delayed graft function
	Zhong [64]	282	DCD	HMP	10.3^c^	SCS	Delayed graft function
	Jochmans [65]	164	DCD	HMP	15.9^c^	SCS	Delayed graft function
	Hosgood [66]	338	DCD	NMP	1	SCS	Delayed graft function (requirement for dialysis in the first 7 days after transplant)

^a^ Intention-To-Treat (ITT).

^b^ Only mean value reported.

^c^ Reported cold ischemia time of perfusion group. Due to absence of oxygenation during perfusion, perfusion time is considered as cold ischemia time.

Abbreviations: N (number), SCS (Static Cold Storage), AST (Aspartate Aminotransferase), EAD (Early Allograft Dysfunction), HOPE (Hypothermic oxygenated perfusion), HMP (Hypothermic Machine Perfusion), NMP (Normothermic Machine Perfusion).

*Ex situ* heart perfusion (ESHP) has progressed from case reports to RCTs and national programs, with PROCEED II demonstrating non-inferiority for NMP vs. static cold storage [49] (Table 1). Later on, case studies [35, 85] and a clinical trial [50] have established DCD hearts as safe alternatives to donation after brain death (DBD) allografts when reanimated and assessed with perfusion platforms, substantially increasing transplant activity without compromising survival [35].

The safety of *ex vivo* lung perfusion (EVLP) was first demonstrated at Lund University Hospital, where the initial six double-lung transplants were successfully performed following re-evaluation on EVLP [86, 87]. Since then, the technique has been developed and implemented clinically [18, 88, 89], with two RCTs confirming safety and efficacy for standard criteria donor (SCD) grafts (Table 1) [51, 52]. Further, the EXPAND and DEVELOP-UK studies transplanted extended criteria donor (ECD) lungs after EVLP, which showed elevated early primary graft dysfunction grade 3 rates, but similar long-term survival compared to standard transplantation of SCD lungs [90, 91]. EVLP has been implemented in many lung transplantation centers across the world for functional assessment of donor grafts as well as increased utilization of ECD lungs [92–98]. However, implementation challenges persist, particularly in smaller-volume centers facing cost and staffing limitations [99–103].

*Ex situ* machine perfusion has been established as an effective strategy to mitigate the deleterious effects of cold ischemia in kidney transplantation. The landmark randomized controlled trial (RCT) by Moers et al. first demonstrated the superiority of hypothermic oxygenated perfusion (HOPE) over static cold storage (SCS), reporting a significant reduction in delayed graft function, improved creatinine clearance, and superior one-year graft survival in the HOPE group [53]. These findings were subsequently corroborated by several independent RCTs, ultimately supporting the integration of routine HOPE into national kidney transplantation programs [104] (Table 1). In contrast, the use of NMP for kidneys has remained mostly experimental. Early clinical studies showed feasibility of kidney transplantation following 1 h of NMP [105, 106]. The first RCT for 1 h NMP of kidneys further demonstrated feasibility and non-inferiority relative to SCS [66]. However, no statistically significant benefit was observed regarding delayed graft function, renal function, or one-year graft survival. Consequently, the role of short-term NMP in kidney transplantation remains a subject of ongoing debate.

Use of NMP for vascularized composite allotransplantation (VCA) has not been adopted for clinical application to the same extent as for solid organs, and clinical cases were only conducted within acceptable limits for warm ischemia. However, small case series and case reports on *ex situ* preservation of amputated extremities and free flaps support *ex situ* machine perfusion as a viable strategy for VCA preservation. This includes reports from Newsome et al. who performed 2.7 h perfusions of (musculo-fascio-cutaneous) anterior thigh flaps with successful transplantation [107], Fichter et al. who perfused radial forearm flaps for 2.5 h, followed by successful transplantation [108], and Taeger et al. who perfused and successfully transplanted a latissimus dorsi flap [109]. Prolonged perfusion durations were further achieved by Taeger et al. who successfully perfused two traumatically amputated lower limbs for 12–16 h at 20 °C [110].

## Long-Term Normothermic Machine Perfusion in Research Settings

While short-term perfusion technologies already introduced benefits upon clinical introduction, they also bring inherent limitations. For example, HOPE approaches profit from lower metabolic activity to prolong preservation by slowing down degradation and simplifying metabolic needs. While HOPE can be used to mitigate IRI and thus recondition the graft [111, 112], it is impossible to treat and repair allografts or perform functional assessment due to the reduced metabolic rate. Therefore, long-term (>24 h) perfusion approaches were developed for different organs and VCAs with the promise to create a platform to treat injured and ECD grafts, perform rigorous graft assessment, and to transition transplant surgeries from an emergency to an elective procedure [13, 15, 113–115]. Further, prolonged perfusion may be beneficial to absorb initial IRI during an acute reperfusion phase on a machine and transplant a fully functional graft once initial inflammation is decreasing again. This hypothesis was formed based on an observation after transplantation of a liver graft after more than 3 days of *ex situ* perfusion [116, 117].

### Liver

In the preclinical setting, *ex situ* perfusion of multiple days was first introduced for livers in 2020 in Zurich [13], demonstrating the feasibility of long-term (i.e., >24 h) preservation. Using a custom-built NMP device, explanted porcine and discarded human livers were preserved for up to 10 days [13]. In contrast to previously known NMP systems, the Wyss–Zurich device allowed for prolonged *ex situ* perfusion in near physiologic conditions and a functional state [13]. Thanks to automation with feed-back controllers, perfusion parameters could be automatically controlled in a tight physiological range, limiting on-site interaction to a minimum. This approach was subsequently validated in the first-in-human application on compassionate use basis, preserving a liver initially declined for transplantation for 3 days followed by successful implantation [116]. The same platform was later adapted to the needs of resected partial livers, which could be used to preserve partial human livers for a week, showing normal tissue integrity and hepatic function [118]. Besides offering a platform for profound viability assessment and organ function, such advancements have since enabled *ex situ* treatment including pharmacological defatting of steatotic grafts [119–121], building on previous efforts in short-term preservation [122, 123]. The first RCT is currently ongoing and data is expected to be available soon (ISRCTN14957538).

Other groups focused on adapting currently available NMP devices to meet the requirements of multi-day perfusion [124–127], i.e., nutrition, precise control of acid-base balance and blood gases, glucose control, as well as dialysis [128–131]. Using and modifying commercially available devices comes with the obvious advantage of easy accessibility and reproducibility [132]. The limitation to this approach is the need for constant human intervention. The longest preservation time with this strategy so far was reported by the Italian group, which successfully preserved a declined human liver over 17 days by incorporating an extracorporeal blood purification system into their NMP device [127]. Indeed, hemodialysis was shown to further improve perfusate quality in multiple organs for long-term perfusions [133]. Further, successful prevention of microbial contamination [134, 135] and hemolysis [13] were found to be crucial for long-term perfusions. Besides mere preservation of viability, the Australia group pioneered the ability to perform liver split procedures of 10 whole livers without interrupting perfusion [126, 136]. Their work is based on the seminal work of performing split procedures during short-term perfusion [137–140] and marks a milestone in *ex situ* liver research. Establishing such long-term perfusion liver models has the immense potential to revolutionize the study of liver injury, repair and regeneration [141–143].

### Heart

While *ex situ* heart perfusion has not been performed for longer than 24 h to date, an increasing amount of case reports that document prolonged *ex situ* perfusions that enabled long distance transport of allografts [144–146]. Notably, a heart was successfully transported across the Atlantic ocean while being perfused *ex situ* for 16 h, illustrating that NMP can even enable world-wide organ sharing [145]. Collectively, the clinical literature agrees on three consistent conclusions: *i.* NMP is safe and non-inferior to SCS for standard donors; *ii.* NMP enables reliable functional assessment that can rescue marginal or DCD hearts; and *iii.* scaling DCD programs with perfusion platforms can substantially expand transplant activity, especially with prolonged perfusion durations that enable longer transport thus wider organ sharing.

### Lung

Extending the interval between donor lung procurement and implantation has several important clinical and logistical implications. Prolonged preservation facilitates broader donor-recipient matching across larger geographic regions and allows transplant centers to avoid nighttime surgery, which is associated with increased complication rates and inferior outcomes [147, 148]. More importantly, lengthening EVLP duration transforms the platform from a short-term assessment tool into a therapeutic environment in which injured or initially discarded lungs can be actively rehabilitated [149].

The Toronto lung transplantation program first reported long-term perfusion in porcine lungs and human discarded donor lungs only years after the advent of EVLP [150]. Ever since, the group has extensively investigated prolonged EVLP in porcine models, demonstrating that continuous EVLP for 12–24 h is feasible [151, 152]. Similarly, other institutions report successful extension of porcine EVLP durations for up to 24 h [153–156], the Hannover program transplanting the extended EVLP lungs into healthy porcine recipients with subsequent short periods of graft evaluation [157]. In Minnesota, the 24 h prolonged evaluation was extended to human discarded donor grafts [158]. A maximum preservation time of 3 days has been reported in porcine lungs using a protocol of two short (4 h) normothermic EVLP cycles alternating with cold storage at 10 °C [159].

Despite strong interest in the topic and several pre-clinical reports of long-term EVLP for up to 24 h or more, clinical evidence remains largely limited to case reports with the longest documented clinical normothermic continuous EVLPs being 11.25 h and 15.5 h, respectively [160, 161]. However, a recent report from the Netherlands group describes the first clinical experience with n-EVLP–HOPE, using hypothermic oxygenated machine perfusion (HOPE) after a period of normothermic EVLP (n-EVLP) in a small cohort of human lung transplantation patients [162]. Grafts from the n-EVLP–HOPE group did not differ significantly in early post-transplantation outcomes compared with the control group.

There are ongoing controversial discussions about what parameters are needed for long-term EVLP, such as what temperature to use, perfusate composition, whether the perfusate should be exchanged in the EVLP and in what intervals. However, despite those advances, additional comprehensive research is needed to advance clinical implementation.

### Vascularized Composite Allografts

Use of machine perfusion has been reported *in vivo* to salvage free flaps after thrombosis of vascular anastomosis. Wolff et al. first reported manual rhythmic perfusion of 3 fibula flaps with heparinized red blood cells at 38 °C for 10–12 days, resulting in flap survival and neovascularization [163]. The same group reported using a closed-loop, low-flow circuit (Novalung MiniLung) at 37 °C, to perfuse thin anterolateral thigh flaps and radial forearm flaps for 4–6 days *in vivo* in five high-risk patients. Stable coverage was achieved in four of five cases. One subtotal flap was lost due to infection, two cases due to complete epithelial loss, and one case featured a venous congestion [164]. Since then, there has been a decade of incremental experimental progress, but NMP of VCAs beyond 24 h remains uncommon [114, 165–167], with most experiments failing by 30 h of perfusion [15, 166, 168]. The longest reported perfusion durations were 72 h for human upper extremities [169] and 144 h for human fasciocutaneous flaps [170].

### Kidney

Similar to liver transplantation, prolonged NMP promises safe inclusion of additional kidney grafts for transplantation. Therefore, protocols and perfusion devices have been refined to extend preservation times beyond 1 day. Notably, the first clinical report was recently published by Dumbill et al. who perfused kidneys for up to 24 h before transplantation and showed correlation between NMP biomarkers and 12-month graft function [171]. Discarded human kidneys could be further perfused for 48 h, while maintaining urine excretion [172]. The longest *ex situ* kidney preservation was reported by de Haan et al. who perfused human kidneys for 4 days at sub-normothermic conditions, maintaining a metabolically active state [173].

### Limitations

Despite recent advances, substantial differences persist in achievable preservation periods across graft types. While organ-specific factors contribute to these variations, maintaining perfusate quality represents a fundamental limitation that constrains perfusion duration across all graft types. The longest perfusion durations were currently achieved for liver grafts, ranging up to 2 weeks [119, 127, 132]. This achievement reflects the liver’s unique capacity to actively maintain perfusate quality through its inherent metabolic and detoxification functions. While soluble waste products and toxins can be easily removed with hemodialysis [133], many metabolic byproducts rely on hepatic clearance. Thus, only perfusate exchange can effectively mitigate waste and toxin accumulation for non-liver grafts.

Maintenance of adequate oxygen delivery presents another critical challenge related to perfusate quality. Erythrocyte supplementation has proven essential for NMP of kidneys, VCAs, livers, and hearts [39, 49, 66, 114]. Even in lung perfusion, where acellular Steen solution is routinely utilized in some EVLP protocols, erythrocyte supplementation has demonstrated improved tissue preservation through enhanced oxygen transport and reduced reactive oxygen species generation [86, 157, 174]. However, hemolysis resulting from shear forces in tubing and pumps, combined with suboptimal environmental parameters [175], necessitates ongoing erythrocyte supplementation to maintain sufficient hematocrit levels.

Beyond waste products and oxygen carriers, perfusate further carries a variety of signaling molecules. Suboptimal management of IRI, inflammatory activation triggered by unphysiological environmental parameters, and endothelial injury induced by oncotic pressure fluctuations, supraphysiological shear forces, and IRI itself collectively limit perfusion duration and initiate release of pro-inflammatory molecules [176, 177]. Although cytokine filters have been employed clinically in kidney, liver, and lung, as well as experimental heart perfusion [178–181], extended perfusion periods require comprehensive pharmacological interventions to minimize IRI and attenuate cellular damage responses.

## Future Impact of Long-Term Preservation

### Graft Assessment

While donor characteristics, such as age or cause of death, as well as procurement parameters, including warm ischemia time, are routinely collected it is challenging to base transplant decisions on these parameters alone [182]. Therefore, machine perfusion has been increasingly used as a platform to perform rigorous graft assessment. As there is no consensus on assessment parameters, we summarized a collection of assessment parameters across different graft types, categorized based on invasiveness and evaluation time [113] (Table 2). Importantly, metabolic function, and associated biomarkers are temperature dependent, leading to higher values for NMP compared to HOPE. For example, the prominent biomarker for mitochondrial injury, flavin mononucleotide (FMN), was established for HOPE in liver transplantation [29–31, 218], but requires different thresholds during NMP [30, 219]. Importantly, many assessment parameters may not be diagnostically conclusive during the first hours of reperfusion as the graft is exposed to IRI and undergoes a temporary reperfusion phase [117]. Hence, all assessment parameters must be evaluated in the context of time, which is currently not standard practice [113]. Another major limitation of current assessment strategies is the lack of normalization for perfusate-borne biomarkers, which should be normalized by perfusate volume and graft size. Given this, transplant communities ideally transition to systematic reporting that allows for comprehensive data collection and identification of indicative assessment parameters with corresponding thresholds, which can be later implemented in national guidelines. Given these, NMP is a platform that substantially improves objective graft assessment and has the potential to safely allow for transplantation of additional grafts.

**TABLE 2:** Collection of assessment parameters from literature classified by the Zurich assessment approach—based on assessment time and invasiveness [113].

Graft type	Stage 1 Perfusion parameters	Stage 2 Graft function (metabolic and mechanistic function)	Stage 3 Organ damage (tissue inflammation and damage markers, imaging modalities)	Stage 4 Invasive testing (biopsy-based analysis)
Liver	• Oxygen consumption [118, 126] • Arterial resistance [126, 183] • pH [39]	• Lactate clearance [39, 40] • Bile production [13] • Ammonia clearance [13] • Bilirubin levels [13, 126] • Glucose metabolism [13] • Albumin synthesis [183] • Coagulation factor synthesis [13]	• AST, ALT [184] • ALP [13, 126] • FMN [29–31, 185, 186] • IL-6 [13] • GGT [126] • LDH [183]	• Steatosis [122, 187] • ATP [13] • Glycogen storage [13] • H & E staining [13, 141] • Gene expression [188]
Heart	• Coronary resistance [189] • Coronary flow [189] • Oxygen consumption [190]	• Lactate [191] • Uric acid [192] • Cardiac high-energy phosphates [192] • Electrograms [191]	• Echocardiography [193] • X-ray fluoroscopy [192] • IL-6 [192] • TNF-α [192] • Heart-type fatty acid binding protein [192] • Procalcitonin [192] • Cardiac troponin [193]	• H&E staining [189] • ATP [194] • TUNEL staining [195] • Caspase-3 staining [195]
VCA	• Graft weight [17] • Vascular resistance [114] • Tissue oxygen saturation [114]	• Lactate [114] • Muscle contractility [114] • Glucose consumption [114] • Lactic acid [114]	• ICG angiography [114] • Thermal imaging [196]	• H&E staining [114] • Caspase-3 staining [196]
Lungs	• Lung oxygenation [197–201] • Pulmonary vascular resistance [197–200] • Pulmonary artery pressure [51, 201, 202] • Perfusion flow [51] • Peak airway pressure [51, 52, 201–203] • Compliance [197–201] • Perfusate loss [201]	• Base excess [201] • Glucose consumption [201] • Lactate production [201] • pH [203, 204]	• IL-6 [205] • IL-8 [205] • NETs [198, 206] • TNF-α [207] • IL-1β [207] • cfDNA [208] • Radiography [201, 209] • Bronchoscopy [201, 209] • Pulmonary artery angioscopy [210] • Ultrasound [211, 212] • Lung weight [213]	• H&E staining [197–200] • Immunohistochemistry staining [197, 198] • Immunofluorescence staining [198] • Mass spectrometry [199, 214] • RNA sequencing [215]
Kidney	• Pressure and flow in renal artery [66, 171, 172, 216] • Macroscopic appearance [66] • Oxygenation [171, 216] • Intrarenal resistance [171, 216] • pCO2 [171, 216]	• Urine production [66, 171, 216] • Urine chloride [172] • Urine potassium [172] • Proteinuria [172] • pH [171, 172, 216] • Lactate [171, 172, 216] • Glucose [171, 172, 216] • GFR [171, 217]	• AST [172] • LDH [171, 172] • CXCL10 [172] • TFF3 [172] • NGAL [171, 172, 216] • Osteopontin [172] • Cystatin C [172] • Clusterin [172] • IP-10 [172] • KIM-1 [216] • TNF-a [172] • VEGF [172] • IL-2, IL1b,IL-4,IL-10,IL-5 [172] • IFN-gamma [172] • L-FABP [171] • Gluthathione Serum transferase [171]	• H & E Staining [171, 172, 216] • Periodic acid Schiff Staining [171] • KIM-1 antibody staining [216] • LC-MS protein quantification [217]

### Graft-Specific Repair Strategies

#### Liver

Multi-day preservation of livers in a functioning state ultimately opens a therapeutic window and provides a platform for therapeutic intervention. Such interventions may be of pharmacological nature, including cell-based or gene therapies, or may be based on novel tissue- and bioengineering approaches [12, 113]. While many strategies have emerged to safely increase the donor pool, we highlight the frequently discussed concepts: defatting of steatotic grafts and regeneration of partial grafts.

##### Defatting of Steatotic Grafts

Grafts with more than 30% macrosteatosis are usually discarded for transplantation because of the established risk of post-transplant liver failure [220–222]. Consequently, reversing hepatic fat infiltration during *ex situ* preservation represents an attractive application of long-term perfusion platforms. Perfusion for 10 days alone can achieve complete defatting in some grafts [119]. Fat metabolization can be further enhanced through pharmacological intervention by leveraging adipose triglyceride lipase (ATGL) driven lipolysis and β-oxidation (L-carnitine, UCB9608, fenofibrate) [119, 120]. These preliminary findings strongly encourage further refinement of protocols enabling efficient and consistent long-term defatting. Other groups found a 40% fat reduction within 6–12 h of NMP with the application of a *defatting cocktail* (forskolin, scoparone, nuclear-receptor ligands, hypericin, and visfatin). Such cocktails, however, raise some concerns regarding toxicity and are not safe for human use [122, 184]. While short-term (<24 h) perfusion platforms are currently being trialed for their anti-fat effects [187], long-term perfusion uniquely opens new horizons to fully and safely reverse clinically relevant steatosis via the addition of pharmacological agents to the perfusate.

##### Liver Regeneration

Achieving *ex situ* regeneration to augment transplantable mass could revolutionize transplant medicine, but relevant volume increase requires sufficient time for tissue proliferation. Major liver surgery is based on the unique hepatic capacity to regenerate. The most remarkable volume increase is seen after ALPPS (Associating Liver Partition and Portal Vein Ligation for Staged Hepatectomy), demonstrating that the human liver can regain up to 80% volume within a week [223, 224]. The pathway responsible for the accelerated regeneration that is seen after ALPPS has been identified to involve paracrine JNK1–IHH signaling [225, 226] and could be a suitable target for future modulation. Various other complex pathways, such as Hippo–YAP1 [227–229] and Wnt/beta catenin [230, 231], play key roles in liver regeneration [232] and may be explored additionally as future therapeutic targets. Furthermore, the regenerative capacity of bile ducts was already explored in a recent study during long-term perfusion [141], with promising results published for cholangiocyte organoid repair [233].

#### Heart

For cardiac allografts, repair and modulation techniques are in the early stages of development. For example, DCD hearts were reconditioned after 30 min of warm ischemia time by reperfusing them at hypothermic conditions with histidine-tryptophane-ketoglutarate-N solution, which improved cell swelling and reduced oxidative stress, nitrosative stress and necrosis prior to normothermic reperfusion [234]. Reconditioning entails improvement of both systolic and diastolic function, which are important for transplant outcome, and come with their own challenges. Diastolic relaxation requires good coronary microvascular function as decreased microvascular circulation can lead to diastolic cross-bridge cycling [235]. This motivates research on vascular and microvascular modulation. It was already demonstrated in a porcine model that HOPE, using traditional histidine-tryptophan-ketoglutarate (HTK) solution, improved systolic function. Similar effects were shown for HOPE with HTK-N solution, which is supplemented with protective amino acids and iron chelators [234]. Compared with regular HTK solution, HTK-N was more effective in improving diastolic function and restoring coronary microvascular circulation (CMVC) [236].

Another *ex situ* treatment is senotherapy, which addresses the adverse effects of aged, senescent cells, such as the senescence-associated secretory phenotype (SASP) in tissues. Use of hearts that were donated from aged donors promises to increase the number of transplantable grafts after senotherapy. Therefore, a first implementation of senotherapy was shown in a rat model during *ex situ* NMP [237]. Here, senomorphic treatment of the donor hearts improved CMVC in grafts substantially during *ex situ* NMP, especially in grafts that were donated from old male animals [237]. As for the other organs and VCAs, only NMP allows for active metabolism, which is required for some repair strategies.

#### Lung

Like other solid organ and VCA perfusion approaches, machine perfusion of lungs offers a unique treatment opportunity for injured donor lungs. Because the organ is isolated during perfusion, therapies can be applied without the risk of systemic off-target effects that would occur if the same treatment were administered *in vivo* [18, 149, 238].

Several interventions have been developed and tested to enhance graft recovery during EVLP and subsequently evaluated during transplantation in pigs. Infected grafts have been successfully treated using cytokine adsorption devices to reduce inflammatory burden and improve lung physiology [149, 197, 214, 239]. Lungs affected by aspiration injury have shown functional recovery following the application of neutrophil extracellular trap (NET) removal technologies [198, 206]. Additionally, mesenchymal stromal cells (MSCs) have been administered during EVLP to stabilize the endothelial and epithelial barriers, attenuate IRI, and promote alveolar repair [149, 199].

Another strategy is gene therapy, using viral vectors to deliver transgenes for up- or downregulation of specific pathways. Moreover, genome-editing tools such as clustered regularly interspaced short palindromic repeats (CRISPR)-based systems can be used to allow for active gene editing in the tissue *in vivo* [18, 238]. For example, one group used an adenoviral vector encoding human IL-10 to enhance IL-10 expression in porcine lungs during EVLP with subsequent transplantation and demonstrated improved lung function 7 days after gene delivery [240]. They further developed this approach by using CRISPR-associated technologies to activate IL1RN and IL-10 in a rat transplantation model, showing that the gene modifications were successfully induced and retained after transplantation into healthy recipients [241]. The Hannover group instead used lentiviral vectors carrying shRNA sequences downregulating swine leukocyte antigen (SLA) to genetically engineer miniature swine donor lungs during EVLP [242]. Remarkably, five of seven treated pigs survived for more than 4 years without immunosuppression, whereas no animals survived in the control group [243].

Another important consideration is the need to ensure that the engineered grafts maintain their function post-transplantation by transplanting EVLP-treated lungs into a relevant animal model for evaluation. As the Lund group and others have shown, lungs treated with, for example, cytokine adsorption or stem cells during EVLP, can deteriorate after transplantation and require additional treatment beyond the EVLP period [197, 199].

As genome-editing and gene-delivery strategies become more complex, longer perfusion times will likely be required. Extended perfusion would allow sufficient time for cellular uptake of the vectors, expression or editing of the target genes, and verification of successful modification before transplantation.

#### Vascularized Composite Allografts

There is currently no evidence supporting specific interventions that can actively promote repair in VCAs. Nevertheless, *ex situ* perfusion appears to exert an intrinsic reconditioning effect, providing the muscle with a physiologic environment that supports cellular repair and functional recovery. The Cleveland group showed that NMP of human upper extremities improved limb condition. At the time of procurement, most (8/10) upper extremities were harvested edematous and cold. However, during the first hours of perfusion, electrolytes, muscle, and surface temperature normalized, and, most importantly, muscle contraction was restored and maintained for 30.5 h [114]. Similarly, the Michigan group demonstrated sustained muscle contractility (grade 4/5) in human limbs during 24 h of perfusion [165].

The Cleveland group performed genomic analysis and identified 2,283 differentially expressed genes in perfused limbs compared to SCS. The perfusion group exhibited upregulation of genes associated with wound healing and inflammation, alongside downregulation of genes involved in apoptosis. These findings suggest that *ex situ* perfusion induces a state of preconditioning that preserves the metabolic viability of VCAs and promotes intrinsic tissue repair mechanisms. Metabolic profiling of perfused human limbs further demonstrated that the tissues remained metabolically active throughout the duration of perfusion over multiple days. Notably, there was a depletion of taurine, an amino sulfonic acid essential for maintaining mitochondrial respiratory chain function. These findings suggest that taurine supplementation during perfusion may mitigate oxidative stress and help preserve mitochondrial integrity [244]. Given these findings, we see substantial potential for future research to establish strategies to enhance muscle repair and regeneration during NMP.

## Conclusion

Short-term machine perfusion has already improved the utilization of donated allografts and is increasingly being adopted in transplant centers worldwide. Beyond enabling DCD heart transplantation and reducing non-anastomotic complications from IRI in liver grafts, perfusion systems have proven valuable for assessing the viability and function of extended-criteria donor organs. However, the maturity of evidence varies considerably across graft types. For VCAs in particular, consistent outcomes have not been achieved yet, even for short-term perfusions with durations between 12 and 24 h, requiring establishment of robust protocols and harmonized outcome reporting as the direct next step.

Beyond short-term use of NMP, growing evidence further suggests that prolonged, multi-day perfusion may support the safe recovery of injured or diseased grafts across a wide range of organ types. With the exception of hearts, multi-day perfusions have now been successfully demonstrated for livers, kidneys, lungs, and vascularized composite allografts, underscoring a strong forward trajectory in the field. Given these predominantly pre-clinical advancements, the clinical utility of long-term perfusion must now be established for livers, lungs, kidneys and hearts through prospective, controlled trials that extend beyond case reports. Such trials are essential not only to develop and validate standardized, organ-specific viability criteria, but also to determine whether prolonged *ex situ* perfusion can effectively mitigate and absorb IRI on the device. Importantly, these investigations must proceed in close collaboration with device manufacturers as part of rigorous regulatory certification processes, as currently no perfusion platform is approved for extended use beyond 24 h. Translation of this technology from experimental application to routine clinical practice further requires logistical frameworks, which may include centralization of national perfusion and repair centers, and legal frameworks for policy development and routine implementation.

Long-term systems also provide a powerful research platform for the discovery and testing of new therapeutic strategies which can be explored in research settings while long-term perfusion is clinically established. Although many of these innovations remain in early development, they hold tremendous promise for enhancing graft utilization and improving transplant safety through rigorous *ex situ* assessment.

Taken together, long-term perfusion represents a promising technological advancement that may translate into broader clinical practice in the near future as the evidence base is continuously maturing. With this transition, we will see a transformation of transplantation logistics and procedures, improved safety for patients, and better utilization of available grafts, ultimately resulting in lower waitlist mortality.
